# Practical Departments

**Published:** 1903-04-04

**Authors:** 


### PRACTICAL DEPARTMENTS.

### HOSPITAL FURNITURE.

Messrs. Atkinson and Co., of Westminster Bridge Road, have a most varied and complete collection of furniture for hospitals and kindred institutions, and their warehouse should be visited by those who have selections to make. They make a speciality of asylum beds, which are strong, neat, and simple in design, and for this latter reason several of them might be utilised with advantage in hospitals also. They are quite moderate in price. The beds are supplied with "Lawson Tait" and other kinds of spring matresses. There are also several very good beds of special design with high sides, for epileptic patients. We are especially struck by a neat and convenient cubicle bedstead, by the use of which any room can be divided into compartments without structural alteration. The device is so simple and inexpensive that we feel its adoption should be universal in institutions where a sleeping apartment has to serve for more than one individual. Messrs. Atkinson show a variety of neat and inexpensive lockers, medicine cupboards in all sizes, with and without glass doors (and some having test-tube fixtures), serviceable ward cupboards, tables, carving tables, dinner waggons and trolleys, wheeling baths, bed rests, bed tables, screens, carrying chairs, and, in fact, all the furniture and fittings for hospitals and kindred institutions. They have amongst household supplies a selection of useful combination furniture suitable for the rooms of the staff of any institution, and where it is necessary to economise space. A glance at Messrs. Atkinson's catalogue is enough to show that they are possessed of much intelligence, and we commend them therefore to the notice of those who desire to supply an institution with appointments.

